# Genetic analysis of intestinal polyp development in Collaborative Cross mice carrying the *Apc*^*Min/+*^ mutation

**DOI:** 10.1186/s12863-016-0349-6

**Published:** 2016-02-19

**Authors:** Alexandra Dorman, Daria Baer, Ian Tomlinson, Richard Mott, Fuad A. Iraqi

**Affiliations:** Tel-Aviv University, Tel-Aviv, Israel; University of Oxford, Oxford, UK

**Keywords:** *Apc*^*Min/+*^, Colorectal cancer, Collaborative cross, Familial adenomatous polyposis, Genetic modifier, *Mom*s, Phenotyping, Recombinant Inbred lines

## Abstract

**Background:**

Colorectal cancer is an abnormal tissue development in the colon or rectum. Most of CRCs develop due to somatic mutations, while only a small proportion is caused by inherited mutations. Familial adenomatous polyposis is an inherited genetic disease, which is characterized by colorectal polyps. It is caused by inactivating mutations in the *Adenomatous polyposis coli* gene. Mice carrying and non-sense mutation in *Adenomatous polyposis coli* gene at site R850, which designated *Apc*^*R850X/+*^ (*Min*), develop intestinal adenomas, while the bulk of the disease is in the small intestine. A number of genetic modifier loci of *Min* have been mapped, but so far most of the underlying genes have not been identified. In our previous studies, we have shown that Collaborative Cross mice are a powerful tool for mapping loci responsible for phenotypic variation. As a first step towards identification of novel modifiers of *Min*, we assessed the phenotypic variation between 27 F1 crosses between different Collaborative cross mice and C57BL/6-*Min* lines.

**Results:**

Here, C57BL/6-*Min* male mice were mated with females from 27 Collaborative cross lines. F1 offspring were terminated at 23 weeks old and multiple phenotypes were collected: polyp counts, intestine length, intestine weight, packed cell volume and spleen weight. Additionally, in eight selected F1 Collaborative cross-C57BL/6*-Min* lines, body weight was monitored and compared to control mice carry wildtype *Adenomatous polyposis coli* gene. We found significant (*p* < 0.05) phenotypic variation between the 27 F1 Collaborative cross-C57BL/6-*Min* lines for all the tested phenotypes, and sex differences with traits; Colon, body weight and intestine length phenotypes, only. Heritability calculation showed that these phenotypes are mainly controlled by genetic factors.

**Conclusions:**

Variation in polyp development is controlled, an appreciable extent, by genetic factors segregating in the Collaborative cross population and suggests that it is suited for identifying modifier genes associated with *Apc*^*Min/+*^ mutation, after assessing sufficient number of lines for quantitative trait loci analysis.

**Electronic supplementary material:**

The online version of this article (doi:10.1186/s12863-016-0349-6) contains supplementary material, which is available to authorized users.

## Background

Genome-wide association studies (GWAS) have identified numerous common alleles that affect cancer susceptibility [1–4], yet the combined effects of the alleles currently discovered are too small to explain the bulk of heritable disease risk. This suggests, that the genetic influence on cancer susceptibility might not be entirely due the combined additive effects of individual alleles. Experimental mouse models of cancer are ideal for examining the effects of genetic modifiers that act epistatically with known susceptibility loci. Epistasis is difficult to detect in human GWAS due to the very large sample size required. However, it is straightforward to engineer mutant mice in which a known susceptibility locus is altered to change the risk of disease. By crossing the mutant onto a population of mice with different genetic backgrounds of naturally occurring variation, it is theoretically possible to map modifier loci.

The great majority of colorectal cancers (CRCs) develop from adenomas [5, 6]. *Apc* mutations are found in the germ line of patients with Familial Adenomatous Polyposis (FAP) and in over 80 % of sporadic colorectal adenomas [7–11]. *Apc* is a tumor suppressor gene [7], that encodes a large protein [8] that regulates Wnt pathway activity through its interaction with the transcription factor β-catenin [12–17]. *Apc* also interacts with numerous actin and microtubule-associated proteins [17–19]. Roles for *Apc* in cell migration have been demonstrated *in vitro* and in mouse models [13, 20].

Several mouse models with heterozygous germline mutations of *Apc* exist, and all of these develop adenomatous intestinal polyps [21, 22]. Different mutations are associated with different disease severities. For example, *Apc*^*R850X/+*^ (*Min*) mice typically develop ~100 intestinal polyps at 3–4 months of age, whereas the *Apc*^*1322T/+*^ mice develop polyps earlier in life, and the *Apc*^*1638N/+*^ model produces only a handful of polyps at over a year of age [21–24]. Like humans with germline mutations in *Apc*, *Min* mice are predisposed to intestinal adenoma formation, which is influenced by modifier loci carried by different inbred strains. Assessing *Ap*c modifiers in the mouse is usually quantitative by counting numbers of intestinal polyps. The *Mom*1 (modifier of *Min* 1) locus was the first *Apc* modifier to be identified [21, 25]. It encodes secretory phospholipase A2 (*Pla2*), a Paneth cell-specific marker with effects on tumorgenesis that remain unclear [24, 26]. Another modifier, *Mom*2, is an *Atp5a1* mutation that acts by suppressing loss of heterozygosity through cell lethality [14, 27, 28]. Other *Mom* loci have been mapped, but no other genes been cloned [29]. The most comprehensive modifier screen to date in a population of female BXH recombinant inbred mice backcrossed to C57BL/6-*Min* males [29] identified five modifier loci. Given that the genetic backgrounds of inbred strains have profound effects on polyp numbers, further modifiers almost certainly exist. These might affect not only polyp numbers, but other traits such as progression to cancer and location in the bowel [30].

The Collaborative Cross (CC) is a panel of recombinant inbred (RI) strains derived from a genetically diverse set of eight founder strains: A/J, C57BL/6 J, 129S1/SvImJ, NOD/LtJ, NZO/HiLtJ, CAST/EiJ, PWK/PhJ and WSB/EiJ. It was designed to provide complex trait analysis with greater power than previous approaches [31, 32]. A full description of the CC population under development at Tel-Aviv University is provided in recent publications [33–40]. The key features of the CC in relation to modifier mapping are the very large number of variants segregating in the population (there are over 36 million SNPs) [36], and the relatively high level of recombination present compared to other mouse RI sets (4.4 million SNPs segregate between the founders C57BL/6 J and C3H/HeJ of the BXH mice [41]). Three founders of the CC are wild-derived strains, representing the three different mice subspecies: *Mus musculus castaneus* (CAST/EiJ)*, Mus musculus musculus* (PWK/PhJ) and *Mus musculus domesticus* (WSB/EiJ), which contribute many sequence variants not segregating among classical strains descended from *Mus musculus domesticus* [41–43], and account for the bulk of the variants segregating in the CC. Consequently mapping of quantitative trait loci (QTLs) using the CC often identifies QTLs involving contrasts between the wild-derived strains [37]. By incorporating polymorphisms data from the genome sequences of the CC founders [36], and restricting attention to variants whose differences across the founders are consistent with the pattern of action of the QTL, candidate genes under QTLs can be identified [36, 37, 44–47].

As a first step towards mapping modifiers of *Apc*^*Min/+*^ in CC mice, we assessed polyp-related phenotypes dissection in F1 hybrids of females from 27 CC lines to C57BL/6-*Min* male mice. In this report, we analyze phenotypic variation and the broad sense heritability (H^2^) of these traits.

## Materials and Methods

### Breeding, genotyping and phenotyping F1 CC-C57BL/6-Min mice

The C57BL/6-*Min* and CC mice were provided by the Small Animal Facility at Sackler Faculty of Medicine, Tel Aviv University (TAU). The CC lines were between the 11th and 38th generation of inbreeding by full-sib mating [34, 48]. They were genotyped using the MEGA-MUGA (60 k SNPs) genotyping array, which showed that each line was at least 85 % homozygous. Full details of the development of the CC are given in Iraqi et al. 2008 [34]. All experimental mice and protocols were approved by the Institutional Animal Care and Use Committee of TAU (approval numbers: M-08-075; M-12-024). Institutional Animal Care and Use Committee of TAU adherers to the Israeli guidelines that follow NIH/USA animal care and use protocols. Mice were housed on hardwood chip bedding in open-top cages at the animal facility and were given tap water and rodent chow *ad libitum*. F1-CC-C57BL/6-*Min* mice were monitored daily for their overall health status. Mice which lost 10 % of their bodyweight between two measured points, or over 20 % of their initial bodyweight, or were observed to be suffering (less movement and activity), and based on the consultation with the Veterinarian at the small animal unit, were terminated.

At 4 weeks old, 0.5 cm tail biopsies were collected and DNA isolated by NaOH boiling method [49]. The F1 mice were genotyped by PCR for the *Apc*^*Min/+*^ mutant allele, using primers MAPC-min (TTCTGAGAAAGACAGAAGTTA), MAPC-15 (TTCCACTTTGGCATAAGG), and MAPC-9 (GCCATCCCTTCACGT). For *Apc* wild type alleles, we used primers MAPC-15 and MAPC-9, which amplify 618 bp PCR products, while for the mutant form we used MAPC-min and MAPC-15, which amplify 317 bp PCR products [50]. For later identification each mouse was ear-labelled.

After five months the F1-CC-C57BL/6-*Min* mice were terminated, their small intestine and colon extracted and washed with PBS. The small intestine were divided into three segments (SB1-proximal, SB2-middle and SB3-distal), and the colon was kept as whole and spread over 3 mm paper. The intestines were fixed in 10 % Neutral Buffered Formalin (NBF) for overnight, and stained by 0.02 % methylene blue. The samples were then examined by binuclear. The number and sizes of polyps in each of the four intestinal sub-regions were recorded as described in Rudling et al. 2006 [51]. The total body weight, weight of spleen, weight and length of intestine and the total packed cell volume (PCV) were also measured at the terminal point. Additionally presented number of polyps per centimeter of intestine, which was calculated using the follow formula: Number of polyp per cm = Total number of polyps/Total length of intestine. Spleen weight was normalized to total body weight using the follow formula: Spleen %Weight = (Spleen weight (g)/total body weight (g)) × 100. Intestine weight was normalized to total body weight using the follow formula: Intestine %Weight = (Intestine weight (g)/total body weight (g)) × 100. Blood was collected by heart puncture [52, 53] into hematocrit heparinized capillary by hematocrit centrifuge CM-70 (ELMI). The % packed cell volume (%PCV) was calculated based on formula: (Total plasma (uL) × 100)/total blood volume (uL).

For 40 selected males from 8 F1-CC-C57BL/6 lines (with representation of control (*Apc*^*+/+*^) and affected (*Apc*^*Min/+*^) mice) body weight was monitored along the experiment at 5 time points, at the weaning (8 weeks old), 12, 16, 19 and 23 weeks old.

### Data analysis

Data analysis performed using a statistical software package SPSS version 19. Analysis of variance (ANOVA) was performed to test the differences of polyp’s number, intestinal %weight and length, spleen %weight, %PCV between 27 F1 CC-C57BL/6-*Min* lines, and body weight between 8 F1 CC-C57BL/6 lines. Clustering analysis of total polyps was performed by Duncan’s test. All the results represented as Mean ± Standard Error, if *n* = 1 represented only the value of the phenotype.

Correlation between traits (polyp number, intestinal %weight and length, polyps per cm, %PCV and spleen %weight) was measured by Pearson product-moment correlation coefficient.

Heritability (H^2^) was estimated as the proportion of phenotypic variation explained by differences between F1 CC-C57BL/6-*Min* lines in the ANOVA, i.e. *H*^*2*^ *= V*_*g*_*/(V*_*g*_ *+ V*_*e*_*)*.

## Results

### Breeding

C57BL/6-*Min* males were crossed to females from 27 CC lines, total progeny of 403 F1 CC-C57BL/6 mice were generated as detailed in Additional file 1: Table 1S. We found that 179/403 mice (44.42 %; 91 males and 88 females) were heterozygous for the knockout allele (carried the *Apc*^*Min/+*^ allele) and therefore were F1 CC-C57BL/6-*Min*. The other progeny were wild-type for the *Apc* allele, and are henceforth denoted as F1 CC-C57BL/6. A second set of 40 male mice including 25 carried *Apc*^*Min/+*^ allele and 15 non carrier (control) of 8 CC lines were also generated, separately to study body weigh differences between these two genotypes.

### Body weight monitoring of F1 CC-C57BL/6 lines

**Fig. 1:**
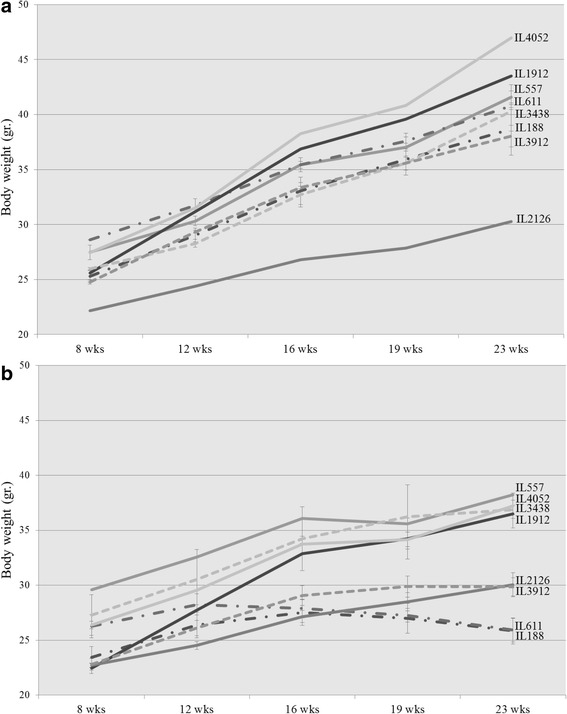
Body weight (±SE) of the **a** control and **b** *Apc* ^*Min/+*^ groups at 5 time points (8, 12, 16, 19 and 23 weeks old) of 8 different set of CC lines. The X-axis represents the time points (in weeks) while the Y-axis represents values of body mass (g). One-way ANOVA performed for statistical analysis, *p* < 0.05

A previous study showed that *Apc*^*Min/+*^ mice lose their body weight during polyp’s development [54, 55]. Here, body weight performances of males from 8 randomly selected F1 CC-C57BL/6-*Min* lines was monitored during the experiment at 5 time points, and compared to the corresponding F1 CC-C57BL/6 wild type lines, results presented in Fig. 1.

At the initial time point (8 weeks old) the body weight of control lines varied between 22.17 g (IL2126) to 28.6 g (IL611) (Fig. 1a). Body weight of *Apc*^*Min/+*^ carriers varied between 22.45 ± 0.46 g (Mean ± Standard Error) (IL1912) to 29.6 g (IL557) (Fig. 1b). One-way ANOVA showed that body weight significantly (*p* < 0.05) different between lines, within both controls and *Apc*^*Min/+*^ groups. In contrast no significant differences were found at the initial time point between the control and *Apc*^*Min/+*^ groups, within each examined line.

During the experiment *Apc*^*Min/+*^ carriers displayed three patterns of body weight changes: body weight loss (marked as - · - · - · - in Fig. 1b), Plateau (---- in Fig. 1b) and continues body weight accumulation ( in Fig. 1b). While all the control lines gained weight continuously during the experiment.

As shown in Fig. 1b, two lines – IL188 and IL611 lost body weight during the experiment. Table 1 shows delta body weight between 23 and 8 wks. old, was -0.34 g and 2.39 g, for IL611 and IL188, respectively. Line IL188 carries *Apc*^*Min/+*^ lost -1.15 g of total body weight between weeks 19 to 23. Over the entire experiment, IL188 mice carries *Apc*^*Min/+*^ gained weight, but started losing weight, only late in the study, whereas IL611 lost weight relative to initial weight. The control mice of same lines gained 12.18 g (IL611) and 13.4 g (IL188), which were significantly different from the *Apc*^*Min/+*^ carriers group. Interestingly, *Apc*^*Min/+*^ carriers from these lines also developed high number of polyps, and will be presented later on.

**Table 1:** Summary of collected phenotypes at 23 weeks old: Total polyps, %PCV, Spleen %Weight and Delta body weight (Body weight 23 weeks old-Body weight 8 weeks old), for control and *Apc* ^*Min/+*^ mice from 8 F1 CC-C57BL/6

CC Line	*Apc*	N (mice)	Total Polyps	%PCV	Spleen %Weight	Delta Body weight (gr.)
IL611	*Min*	3	55.33 ± 10.91	25.96 ± 4.32	2.7637 ± 0.677	-0.34
	WT	2	0	54.62 ± 0.1	0.1962 ± 0.0007	12.18
IL188	*Min*	8	70.38 ± 10.78	29.45 ± 3.8	1.9698 ± 0.3419	2.39
	WT	3	0	54.93 ± 2.53	0.1762 ± 0.0667	13.4
IL3438	*Min*	2	67.5 ± 24.5	38.93 ± 6.67	1.2124 ± 0.691	9.59
	WT	2	0	53.75 ± 4.55	0.1729 ± 0.024	14.36
IL3912	*Min*	3	42.33 ± 5.36	35.2 ± 2.29	0.7768 ± 0.1209	7.03
	WT	2	0	56.62 ± 4.06	0.1699 ± 0.0496	13.28
IL2126	*Min*	4	14.75 ± 4.05	51.75 ± 1.54	0.2966 ± 0.0602	7.33
	WT	1	0	51.92	0.1651	8.11
IL557	*Min*	1	53	34.25	1.4379	8.65
	WT	3	0	55.6 ± 5.92	0.2656 ± 0.0357	14.12
IL1912	*Min*	2	43.5 ± 1.5	41.84 ± 1.84	0.5044 ± 0.0786	14.04
	WT	1	0	53.75	0.1609	17.9
IL4052	*Min*	2	9	48.96 ± 5.38	0.3218 ± 0.017	10.85
	WT	1	0	60.26	0.1703	19.51

The *Apc*^*Min/+*^ carriers group of lines IL3438 and IL3912 (Fig. 1b) exhibited Plateau pattern during the final weeks of the experiment (after week 20). Table 1 shows that delta body weight for these lines was 9.59 g (IL3438) and 7.03 g (IL3912). Control mice from the same lines gained 14.36 g (IL3438), which wasn’t significantly different from *Apc*^*Min/+*^ carriers, and 13.28 g (IL3912) which was significantly different (*p* < 0.01) compared to *Apc*^*Min/+*^ carriers. Values for the rest tested phenotypes of these lines summarized in Table 1.

The remaining lines – IL2126, IL557, IL1912 and IL4052, which are also presented in Fig. 1b, have demonstrated continues increase in body weight throughout the experiment. Line IL2126 has exhibited no significant difference in weight accumulation tendency between the experimental (7.33 g) and control groups (8.11 g). Line IL557 showed significant difference (*p* < 0.039) in delta body weight accumulation between the experimental (8.65 g) and control groups (14.12 g). Line IL1912 showed no significant difference in delta body weight accumulation between the experimental (14.04 g) and control groups (17.9 g). Line IL4052 showed no significant difference in delta body weight accumulation between the experimental (10.85 g) and control groups (19.51 g). Values for the rest tested phenotypes summarized in Table 1.

At the final time point of the experiment (23 wks. old) lines from both groups, control and *Apc*^*Min/+*^ carriers, displayed significant differences in their body weight values. Additionally at this step of the experiment we found significant variation between control and *Apc*^*Min/+*^ carriers group within each line.

### Polyp counts in F1 CC-C57BL/6-Min mice

**Fig. 2:**
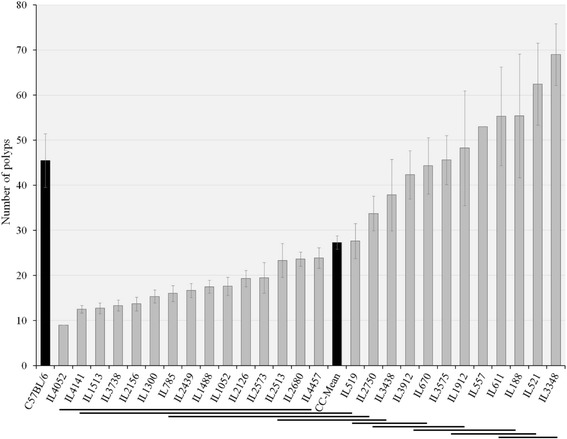
Average number of polyps (±SE) in 27 F1 CC-C57BL/6-*Min* lines (*n* = 1-16 mice/line)*.* The X-axis represents CC lines, C57BL/6 strain carrying the *Apc* ^*Min/+*^ mutation (first black column) and CC-Mean (black column ~ in the middle), while the Y-axis number of polyps. Continuous lines under the graph represent 9 clusters based on Duncan’s Least Significant Range (LSR) test. One-way ANOVA performed for statistical analysis, *p* < 0.05

Mice were terminated at age of 23 weeks and the gastrointestinal tract (small intestine and colon) was extracted for polyps count, results are presented in Fig. 2. Across the 27 F1 CC-C57BL/6-*Min* lines mean polyp count was 27.3 ± 1.49. The cohort exhibited a wide range of phenotypic variability, from 9 polyps (IL4052) to 69 ± 6.83 (IL3348). One-way ANOVA revealed significant variation between CC lines (*p* < 0.01). Polyp counts were approximately normally distributed, suggesting intervention of numerous genetic factors in this trait. Duncan’s analysis divided tested lines into 9 clusters, presented by continuous lines under the graph in Fig. 2. Interestingly, mean polyp count in the parental line C57BL/6-*Min* was 45.50 ± 5.98, so that the majority (20/27, 74.07 %) of F1 CC-C57BL/6-*Min* lines have fewer polyps than the parental line, despite the fact that the number of defective *Apc* alleles is the same in all cases. When we compared the polyp counts in the male mice of the 8 CC lines assessed in the second set of lines presented in Fig. 1a and Table 1 and Additional file 1: Table 7S, majority of lines were consistent on both sets. Notably, that SE is very high in many lines. Based on one way ANOVA, we have found that some lines are significantly different between male and female, mice. As for polyp counts, we have found that some lines are significantly different with this trait between both sexes as presented in Additional file 1: Table 7S, however, overall across all the tested lines sex was significant different with polyp counts.

**Fig. 3:**
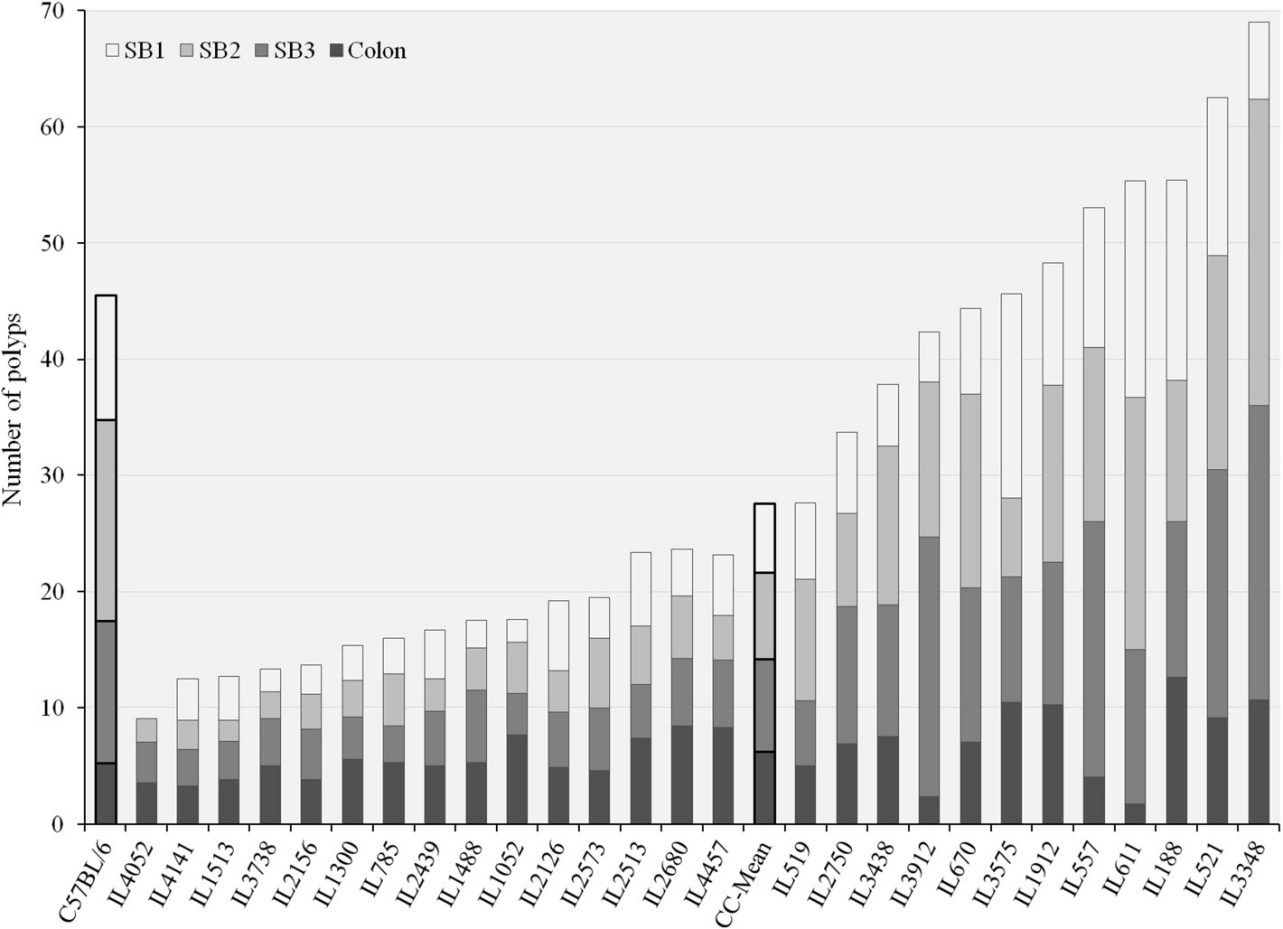
Distribution of all counted polyp in three parts of small intestine: SB1-proximal, SB2-middle, SB3-distal and colon. The X-axis represents CC lines, C57BL/6 strain carrying the *Apc* ^*Min/+*^ mutation (first black column) and CC-Mean (black column ~ in the middle), while the Y-axis number of polyps. One-way ANOVA performed for statistical analysis, *p* < 0.05

Next we asked if different segments of the intestine exhibit differential polyp distribution. The small intestine was subdivided into 3 parts (small intestine proximal-SB1, middle-SB2 and distal-SB3), and the colon was related separately. Polyp counts were recorded in each region. Figure 3 shows the distribution of polyps in the four components of gastrointestinal tract in the 27 F1 CC-C57BL/6-*Min* lines and C57BL/6-*Min*. Overall, polyps were distributed approximately equally between 4 segments: SB1 with 5.96 ± 0.4 polyps (21.58 %), SB2 with 7.73 ± 8.17 (26.98 %), SB3 with 7.97 ± 7.69 (28.97 %) and colon with 6.2 ± 3.9 (22.47 %). The percentages of polyp counts in C57BL/6 J*-Min* strain were 23.63 % (SB1), 37.91 % (SB2), 26.92 % (SB3) and 11.54 % (colon).

Interestingly some lines showed approximately the same number of polyps, which were differentially distributed across the intestine. For example, line IL611 had 55.33 ± 10.91 and line IL188 55.4 ± 13.71 polyps. However, in IL611 only 3.01 % of polyps were in the colon; while IL188 had 22.74 % of polyps in the same segment. Similarly in SB2, IL611 showed 39.16 % polyps and IL188 22.02 %. This suggests that different genetic factors might be involved in polyp development throughout the gastrointestinal tract.

### Intestine Length

Since tumor development might affect the length and weight of the intestine, we asked if these phenotypes also differed across the population. Additional file 1: Figure 1S (presented in supplementary matherials) shows the distribution of intestine length (cm) across the F1 CC-C57BL/6-*Min* lines and the C57BL/6-*Min* parental strain. The mean intestine length of the entire population was 47.92 ± 0.28 cm, ranging from 43.52 ± 0.85 cm (IL2126) to 51.83 ± 1.21 cm (IL3348). One-way ANOVA showed that intestine length varied significantly (*p* < 0.01) between 27 F1 CC-C57BL/6-*Min* lines. The mean intestine length for the parental strain, C57BL/6-*Min*, was 48.55 ± 1.55 cm.

### Number of polyps per centimeter

Next, number of polyps per cm of intestine was calculated (as described in Matirials and Methods section), results presented in Additional file 1: Figure 2S. As for entire population, avarge number of polyps per cm was 0.5676 ± 0.0291. Lowest value of 0.1877 ± 0.0356 polyps per cm was recorded for line IL4052, which had overall only 9 polyps. Highest value of 1.3267 ± 0.0421 polyps per cm was recorded for line IL3348, which had overall 69 ± 6.836 polyps. The parental line C57BL/6 showed 1.142 ± 0.0297 polyps per cm.

Additionally, histogram plot in Additional file 1: Figure 3S shows frequency of number of lines within different ranges of polyps per cm values. It was found that majority of tested CC lines, 9 lines, developed between 0.2721 ± 0.1815 to 0.4143 ± 0.0283 polyps per cm of intestine. As for extreme cases: one line (IL4052) developed 0.1877 ± 0.0356 polyps per cm, and 2 lines (IL521 and IL3348) developed more than 1.2209 ± 0.0177 polyps per cm.

### Intestine Weight

Additional file 1: Figure 4S shows the distribution of intestine %weight*.* The mean across the entire population was 2.63 ± 0.71 %, ranging from 1.63 ± 0.16 % (IL2750) to 4.82 ± 0.71 % (IL611). One-way ANOVA showed that the intestine %weight varied significantly (*p* < 0.01) between tested lines. The mean intestine %weight in C57BL/6*-Min* was 4.5 ± 0.08.

### Packed Cell Volume

*Apc*^*Min/+*^ mice suffer from severe anemia [56], and severe anemia is the main cause of death in these mice [57]. In order to investigate this phenmenon in the F1 CC-C57BL/6-*Min* population, the packed cell volume (PCV) was measured at the terminal point of experiment, results are summarized in Additional file 1: Figure 5S. The overall mean of entire population was 44.6 ± 0.7 %, and individual lines ranged from 25.96 ± 4.32 % (IL611) to 51.94 ± 1.33 % (IL4457). Normal PCV values are in the range 45-55 % [56], so 51.85 % (14/27) of the CC-C57BL/6-*Min* lines were below the normal range. One-way ANOVA revealed that %PCV varied significantly (*p* < 0.01) between F1 CC-C57BL/6-*Min* lines. The parental C57BL/6*-Min* line showed the lowest %PCV value of 24.35 ± 2.13 %. When we compared the %PCV in the male mice of the 8 CC lines assessed in the second set of lines presented in Fig. 1a and Table 1, majority of lines were consistent on both sets. Notably, that SE is very high in many lines.

### Spleen Weight

It has been reported that *Apc*^*Min/+*^ mice suffer from splenomegaly, (enlargement of the spleen) [58]. Additional file 1: Figure 6S shows the distribution of spleen %weights. The mean of the tested 27 F1 CC-C57BL/6-*Min* lines was 0.53 ± 0.05 %, ranging from 0.18 ± 0.02 % (IL3738) to 2.76 ± 0.67 % (IL611). One-way ANOVA showed that spleen %weight varied significantly (*p* < 0.01) between lines. The parental line, C57BL/6-*Min*, had relatively high value of 1.52 ± 0.22 %. When we compared the spleen weight in the male mice of the 8 CC lines assessed in the second set of lines presented in Fig. 1a and Table 1, majority of lines were consistent on both sets. Notably, that SE is very high in many lines.

### Correlation between phenotypes

Next we were interested to investigate the relationship between polyp’s development and related phenotypes. Table 2 shows Pearson correlations between traits across the 27 F1 CC-C57BL/6-*Min* lines.

**Table 2:** Pearson correlation values between traits: total polyps, intestine length, intestine %weight, %PCV, spleen %weight and polyps per cm

	Total Polyps	Intestine Length	Intestine %Weight	%PCV	Spleen %Weight	Polyps per cm
Total Polyps	1	0.589**	0.695**	-0.841**	0.743**	0.998**
Intestine Length	0.589**	1	0.266	-0.457*	0.317	0.539**
Intestine %Weight	0.695**	0.266	1	-0.769**	0.815**	0.714**
%PCV	-0.841**	-0.457*	-0.769**	1	-0.846**	-0.852**
Spleen %Weight	0.743**	0.317	0.815**	-0.846**	1	0.764**
Polyps per cm	0.998**	0.539**	0.714**	-0.852**	0.764**	1

*Correlation is significant at *p* < 0.05 **Correlation is significant at *p* < 0.01

**Fig. 4:**
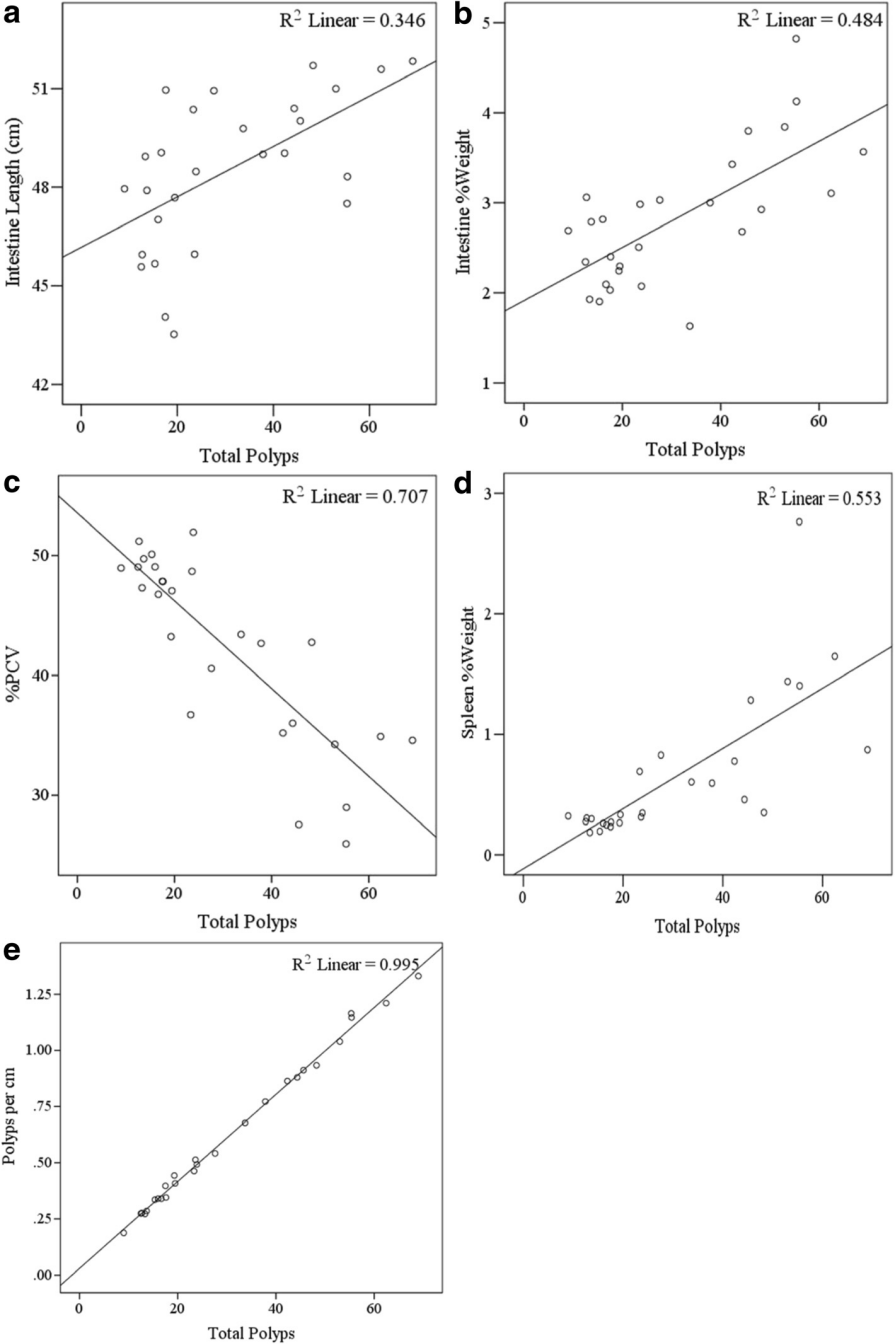
Scatter plot with tread lines of total polyps vs. **a**. Intestine length (R^2^ = 0.346), **b**. Intestine %Weight (R^2^ = 0.484), **c**. % PCV (R^2^ = 0.707), **d**. Spleen %Weight (R^2^ = 0.553) and **e**. Polyps per cm (R^2^ = 0.995)

The length of intestine and total number of polyps is positively correlated (Pearson correlation coefficient (r) = 0.589, *p* < 0.01). This suggests that a high number of polyps are associated with longer intestinal track. The scatter plot in Fig. 4a (R^2^ = 0.346) shows data distribution of these two traits.

Similarly intestine %weight is positively correlated with total polyps count (*r* = 0.695, *p* < 0.01). Suggesting that a high number of polyps are associated with heavier intestinal track. The scatter plot in Fig. 4b (R^2^ = 0.484) shows data distribution of these two traits.

In contrast, a negative correlation (*r* = -0.841, *p* < 0.01) was observed between %PCV and total number of polyps, i.e. high number of polyps are associated with low values of %PCV. The scatter plot in Fig. 4c (R^2^ = 0.707) shows data distribution of these two traits.

The spleen %weight and total number of polyps were positively correlated (*r* = 0.743, *p* < 0.01). This suggests that high numbers of polyps are associated with heavier spleen, indicating splenomegaly. The scatter plot in Fig. 4d (R^2^ = 0.553) shows data distribution of these two traits.

As was expected there is strong positive correlation was found between %PCV and spleen %weight. Pearson test showed negative correlation (*r* = -0.846, *p* < 0.01) between these traits, suggesting that anemia accompanied by splenomegaly.

As was expected there is strong positive correlation between Total polyps and Polyps per cm (*r* = 0.998, *p* < 0.01), corresponding scatter plot in Fig. 4e (R^2^ = 0.995). Additionally significant positive correlations were found between Polyps per cm and Intestine length (*r* = 0.539, *p* < 0.01), Intestine %Weight (*r* = 0.714, *p* < 0.01) and Spleen %Weight (*r* = 0.764, *p* < 0.01), significant negative correlation was found with %PCV (*r* = -0.852, *p* < 0.01).

### Heritability

Our last aim of this repost was to determine whether variation in polyp development and other tested phenotypes has a genetic basis in F1 CC-C57BL/6-*Min* population. In order to answer this question, heritiabilities (H^2^) values were calculated, results summarized in Table 3. All the phenotypes have high heritability values, meaning that tested traits have strong genetic basis. The highest value of 0.641 was found for the phenotype of all counted polyps in along the gastrointestinal tract (Total Polyps), while the lowest value of 0.305 was found for the number of polyps in the colon.

**Table 3:** Mean square between and within groups, and H^2^ calculations for traits of: total number of polyps, polyp’s distribution in different parts of intestine (SB1, SB2, SB3) and Colon, intestine length, polyps per cm, intestine %weight, %PCV and spleen %weight

	Total polyps	SB1	SB2	SB3	Colon	Intestine Length	Polyps per cm	Intestine %Weight	%PCV	Spleen %Weight
MS between	1874.734	124.804	265.143	241.036	40.197	37.665	0.678	2.992	334.972	1.654
MS within	150.509	12.580	32.091	26.832	10.510	8.471	0.059	0.521	43.910	0.202
H2	0.641	0.581	0.530	0.553	0.305	0.349	0.621	0.424	0.508	0.528

## Discussion

This is the first analysis of intestinal polyp’s development and related traits in 27 F1 CC-C57BL/6 lines carrying the *Apc*^*Min/+*^ mutation. All the tested phenotypes showed wide variability, suggesting that the diverse genetic background of the CC lines is contributes to this variation. Analysis of heritability confirmed this statement by showing that the majority of phenotypic variation is due to genetics.

Previous studies have shown that *Apc*^*Min/+*^ mice lose body weight during disease progression [59], which mimics the loss of weight observed in cancer patients, especially of gastrointestinal cancers [60]. In our study, in eight randomly selected lines, we found that body weight at the start of experiment showed significant variation between lines, but there were no significant differences between knockout and control mice within each line. This suggests that initially the presence of *Apc*^*Min/+*^ mutation has no influence on body weight. However, throughout the experiment we identified 3 different patterns of body weight performances in *Apc*^*Min/+*^: loss of body weight, plateau and continuous accumulation of body weight. Resistant lines accumulate their body weight continuously and do not develop multiple polyps, while susceptible lines lose body weight, presumably because of the polyp burden. These results emphasize the importance of genetic background in the response to *Apc*^*Min/+*^ mutation.

Previous studies have shown that genetically diverse mouse strains, carrying *Apc*^*Min/+*^ mutation, vary in intestinal tumor development [29]. Our results support this finding. Additionally, we found that polyp counts in F1 CC-C57BL/6-*Min* lines are distributed below and above the parental (C57BL/6-*Min*) mean value. We conclude that the CC population contains suppressors and enhancers genetic elements of the *Apc*^*Min/+*^ mutation.

The different four parts (SB1, SB2, SB3 and colon) of the gastrointestinal tract have specific physiological function, due to different gene expression profiles, pH and microbiota [61]. In a previous study with *Apc*^*Min/+*^ mice it was reported that the density of polyps varies across the gastrointestinal tract [23]. Our results confirm this observation and suggest that tumorgenesis in different parts of intestine is controlled by different genes. Additionally current results suggest that high levels of polyp proliferation in *Apc*^*Min/+*^ mice affect the length and weight of the intestine. The much polyps mouse have the longer and heavier the gastrointestinal tract.

Our data also support the findings that *Apc*^*Min/+*^ mice suffer from anemia [56] as we observed a negative correlation between the number of polyps and %PCV. Susceptible lines with high total polyp counts had low %PCV values, while resistant lines for polyp development displayed normal %PCV values.

*Apc*^*Min/+*^ mice also develop splenomegaly [59]. Possible causes include viral or bacterial infections, cirrhosis, venous pressure in spleen or liver, metastasis of solid tumors or hemolytic anemia [60]. Our results confirm that *Apc*^*Min/+*^ mice develop splenomegaly concurrently with polyp development. Additionally strong negative correlation was found between the size of spleen and %PCV values. Therefore, we suggest that the main cause for splenomegaly is the hemolytic anemia. Several studies have already showed that erythrocytes trapped in the spleen cause the enlargement of this organ and increase anemia [59].

Finally we conclude that modifiers for *Apc*^*Min/+*^ which affect intestinal tumor development segregate in the CC population, and that these loci are likely to be mappable by future QTL analysis. Unfortunately, the number assessed CC lines in this study (27 lines) were not sufficient and to provide enough statistical power for mapping QTL, and it is believed with more lines (additional 20 or so lines) to be assessed in the future, strong and significant QTL and suggested candidate genes will be achieved.

## Conclusions

Variation in polyp development is controlled, an appreciable extent, by genetic factors segregating in the Collaborative cross population and suggests that it is suited for identifying modifier genes associated with *Apc*^*Min/+*^ mutation, after assessing sufficient number of lines for quantitative trait loci analysis. The expected findings may be used for early prediction of potential intestine cancer development in host carrying susceptible genetic factors, thus can be applied for better control and sufficient apply therapy tools and approaches.

## Availability of Data

All raw collected data which used in this manuscript will be, freely available from http://phenome.jax.org/.

## Additional file

Additional file 1:
**Table 1S:** Summary of all generated progeny, divided by gender & *Min* carriers. **Figure 1S.** Intestinal length (cm) (±SE). The X-axis represents CC lines, C57BL/6 strain carrying the *Apc* ^*Min/+*^ mutation (first black column) and CC-Mean (black column ~ in the middle), while the Y-axis centimeter. One-way ANOVA performed for statistical analysis, *p* < 0.05. **Figure 2S.** Number of polyps per cm (±SE). The X-axis represents CC lines, C57BL/6 strain carrying the *Apc* ^*Min/+*^ mutation (first black column) and CC-Mean (black column ~ in the middle), while the Y-axis is number of polyps. One-way ANOVA performed for statistical analysis, *p* < 0.05. **Figure 3S.** Frequency of lines as for the number of polyps per cm. The X-axis represents number of polyps per cm, while the Y-axis is the frequency. Number inside each column is the number of CC lines within each range of polyps. **Figure 4S.** Intestine %Weight (±SE). The X-axis represents CC lines, C57BL/6 strain carrying the *Apc* ^*Min/+*^ mutation (first black column) and CC-Mean (black column ~ in the middle), while the Y-axis %Weight. One-way ANOVA performed for statistical analysis, *p* < 0.05. **Figure 5S.** %PCV (±SE). The X-axis represents CC lines, C57BL/6 strain carrying the *Apc* ^*Min/+*^ mutation (first black column) and CC-Mean (black column ~ in the middle), while the Y-axis %. One-way ANOVA performed for statistical analysis, *p* < 0.05. **Figure 6S.** Spleen %Weight (±SE). The X-axis represents CC lines, C57BL/6 strain carrying the *Apc* ^*Min/+*^ mutation (first black column) and CC-Mean (black column ~ in the middle), while the Y-axis %Weight. One-way ANOVA performed for statistical analysis, *p* < 0.05. **Figure 7S.** Average number of polyps (±SE) in 22 F1 CC-C57BL/6-*Min* lines (*n* = 1-16 mice/line) based on male and female*.* The X-axis represents male and female of each studied CC line, while the Y-axis number of polyps. (PDF 998 kb)
