# Erratum to: Genetic analysis of intestinal polyp development in Collaborative Cross mice carrying the Apc Min/+ mutation

**DOI:** 10.1186/s12863-016-0456-4

**Published:** 2016-11-24

**Authors:** Alexandra Dorman, Daria Baer, Ian Tomlinson, Richard Mott, Fuad A. Iraqi

**Affiliations:** 1 Tel-Aviv University, Tel-Aviv, Israel; 2 University of Oxford, Oxford, UK

## Erratum

Following publication of this article [1], it came to the author’s attention that the there was some funding information missing from the acknowledgements section. The updated funding information has been included in the original article and can be seen below:

This study was also supported by a Project Grant from the Israel Cancer Research Fund (ICRF) and a grant from Israeli Science Foundations (ISF) number (961/15).

## References

[1] Dorman A, Baer D, Tomlinson I, Mott R, Iraqi F (2016). Genetic analysis of intestinal polyp development in Collaborative Cross mice carrying the Apc Min/+ mutation. BMC Genet.

