# Supplementary material for: Genetic analysis of intestinal polyp development in Collaborative Cross mice carrying the ApcMin/+ mutation
Source: BMC Genet. 2016 Feb 19;17:46. doi: 10.1186/s12863-016-0349-6 (PMC4761170; doi:10.1186/s12863-016-0349-6)

Table 1S: Summary of all generated progeny, divided by gender & *Min* carriers

	Total progeny	Males	Females	Total *Min* carriers	Males	Females
**IL188**	13	7	6	5	3	2
**IL519**	13	6	7	5	3	2
**IL521**	22	15	7	9	7	2
**IL557**	4	3	1	1	0	1
**IL611**	8	2	6	3	0	3
**IL670**	5	3	2	3	3	0
**IL785**	14	8	6	7	5	2
**IL1052**	12	5	7	5	2	3
**IL1300**	31	13	18	15	7	8
**IL1488**	13	8	5	8	5	3
**IL1513**	19	5	14	10	2	8
**IL1912**	9	5	4	4	2	2
**IL2126**	18	7	11	10	4	6
**IL2156**	19	9	10	9	3	6
**IL2439**	13	7	6	6	3	3
**IL2513**	11	7	4	3	3	0
**IL2573**	29	13	16	15	7	8
**IL2680**	12	4	8	5	1	4
**IL2750**	13	10	3	7	6	1
**IL3348**	15	9	6	6	4	2
**IL3438**	14	3	11	6	1	5
**IL3575**	16	9	7	5	2	3
**IL3738**	8	5	3	3	3	0
**IL3912**	8	2	6	3	0	3
**IL4052**	6	1	5	2	0	2
**IL4141**	24	15	9	8	6	2
**IL4457**	34	19	15	16	9	7
**Total**	403	200	203	179	91	88

Figure 1S. Intestinal length (cm) (±SE). The X-axis represents CC lines, C57BL/6 strain carrying the *Apc*$^{Min/+}$ mutation (first black column) and CC-Mean (black column ~ in the middle), while the Y-axis centimeter. One-way ANOVA performed for statistical analysis, p<0.05.

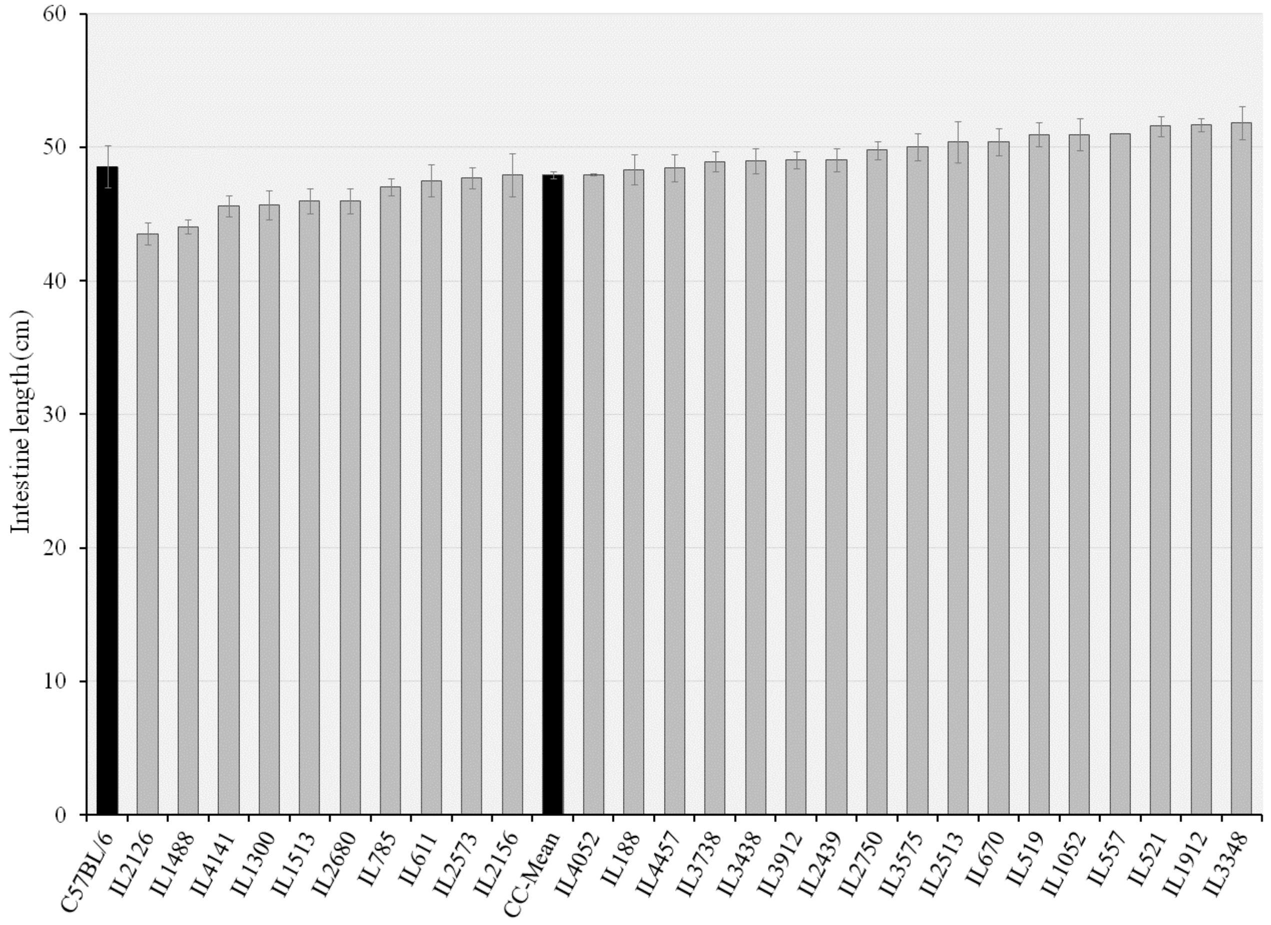

Figure 2S. Number of polyps per cm (±SE). The X-axis represents CC lines, C57BL/6 strain carrying the *Apc*$^{Min/+}$ mutation (first black column) and CC-Mean (black column ~ in the middle), while the Y-axis is number of polyps. One-way ANOVA performed for statistical analysis, p<0.05.

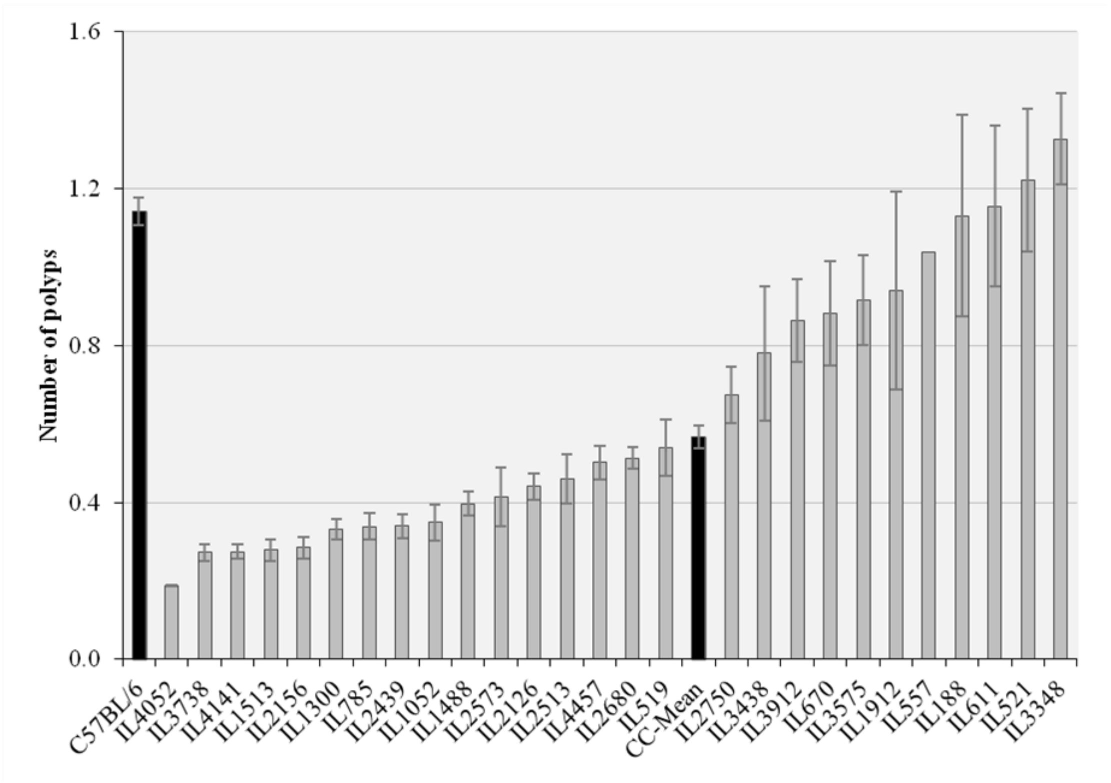

Figure 3S. Frequency of lines as for the number of polyps per cm. The X-axis represents number of polyps per cm, while the Y-axis is the frequency. Number inside each column is the number of CC lines within each range of polyps.

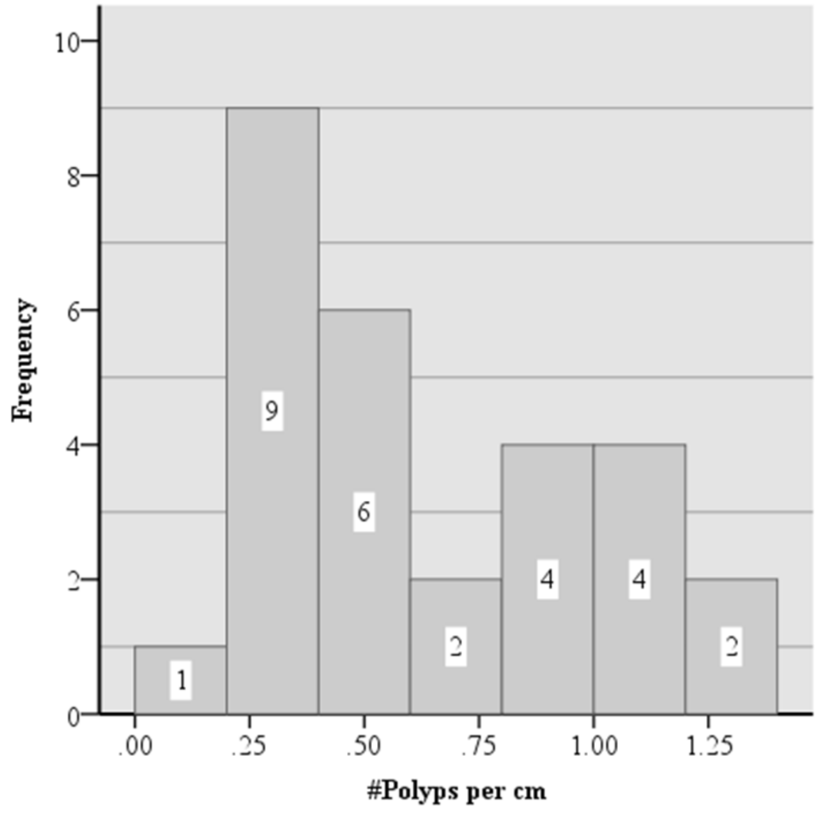

Figure 4S. Intestine %Weight (±SE). The X-axis represents CC lines, C57BL/6 strain carrying the *Apc*$^{Min/+}$ mutation (first black column) and CC-Mean (black column ~ in the middle), while the Y-axis %Weight. One-way ANOVA performed for statistical analysis, $p<0.05$.

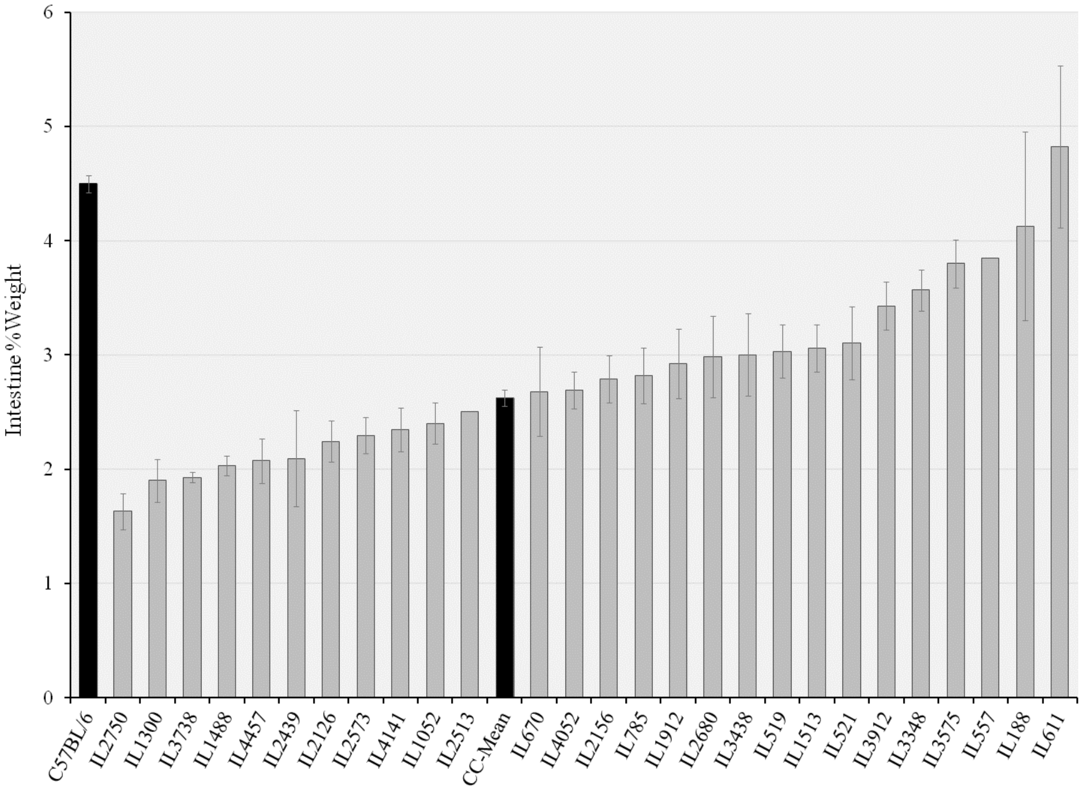

Figure 5S. %PCV (±SE). The X-axis represents CC lines, C57BL/6 strain carrying the *Apc*$^{Min/+}$ mutation (first black column) and CC-Mean (black column ~ in the middle), while the Y-axis %. One-way ANOVA performed for statistical analysis, $p<0.05$**.**

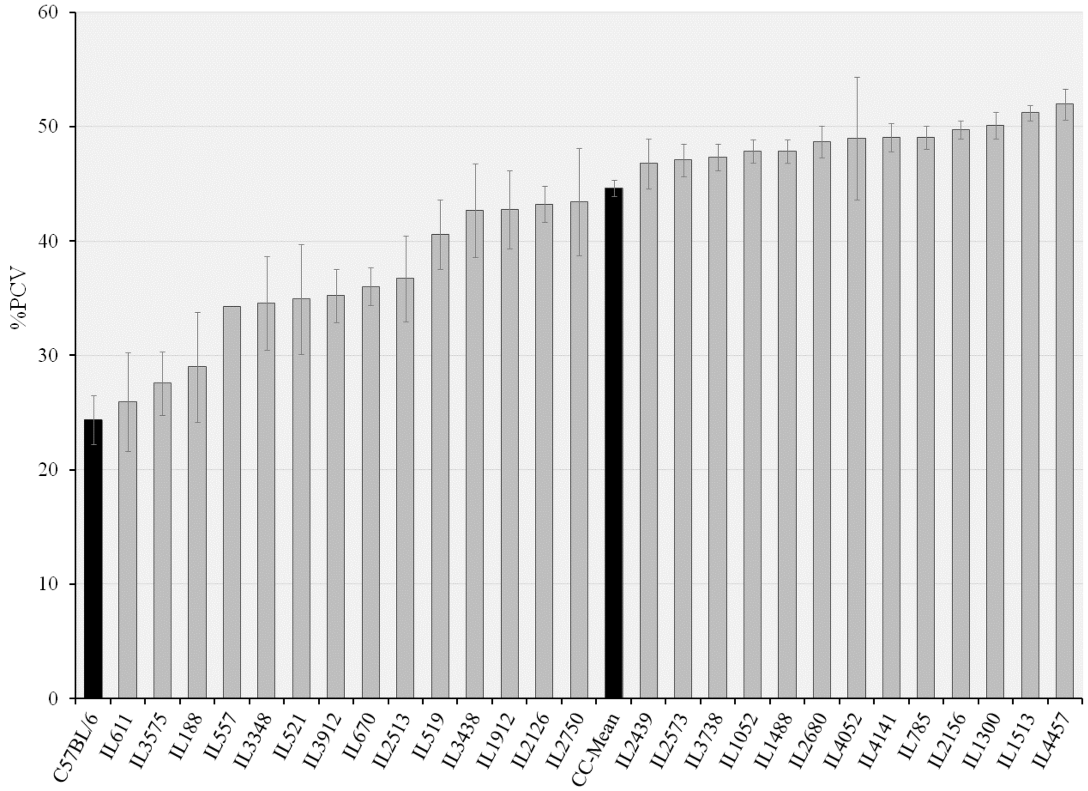

Figure 6S. Spleen %Weight (±SE). The X-axis represents CC lines, C57BL/6 strain carrying the *Apc*$^{Min/+}$ mutation (first black column) and CC-Mean (black column ~ in the middle), while the Y-axis %Weight. One-way ANOVA performed for statistical analysis, p<0.05.

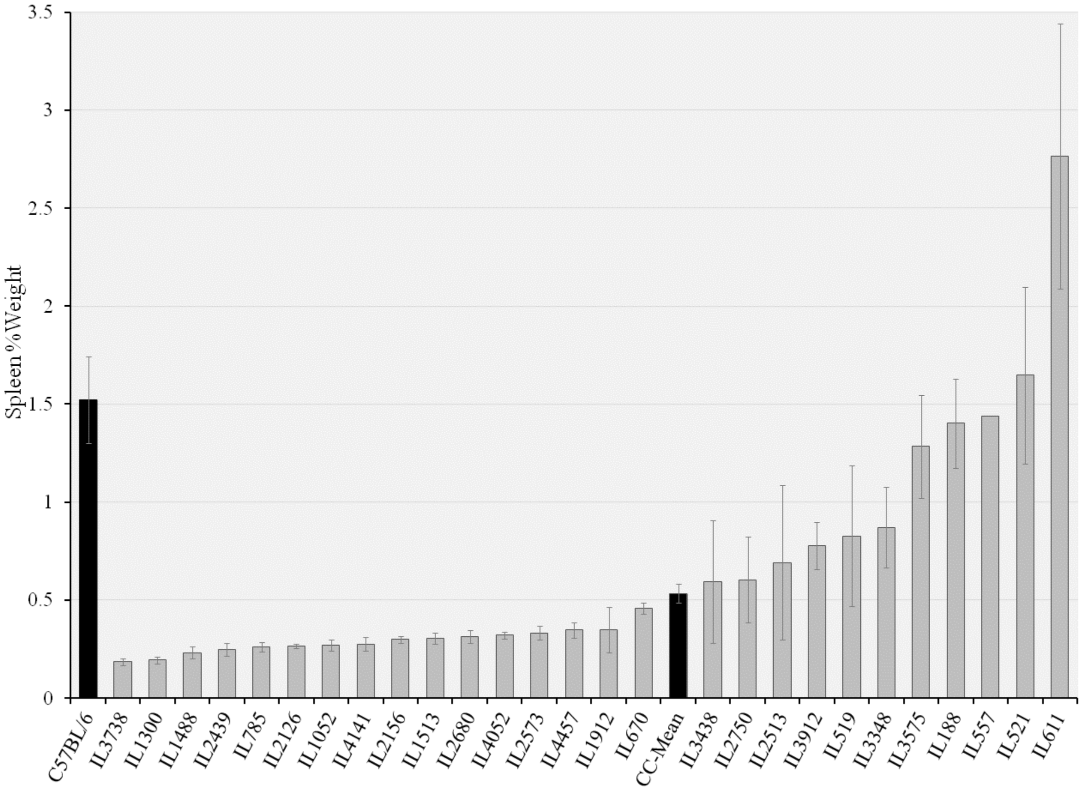

Figure 7S. Average number of polyps (±SE) in 22 F1 CC-C57BL/6-*Min* lines (n= 1-16 mice/line) based on male and female. The X-axis represents male and female of each studied CC line, while the Y-axis number of polyps.

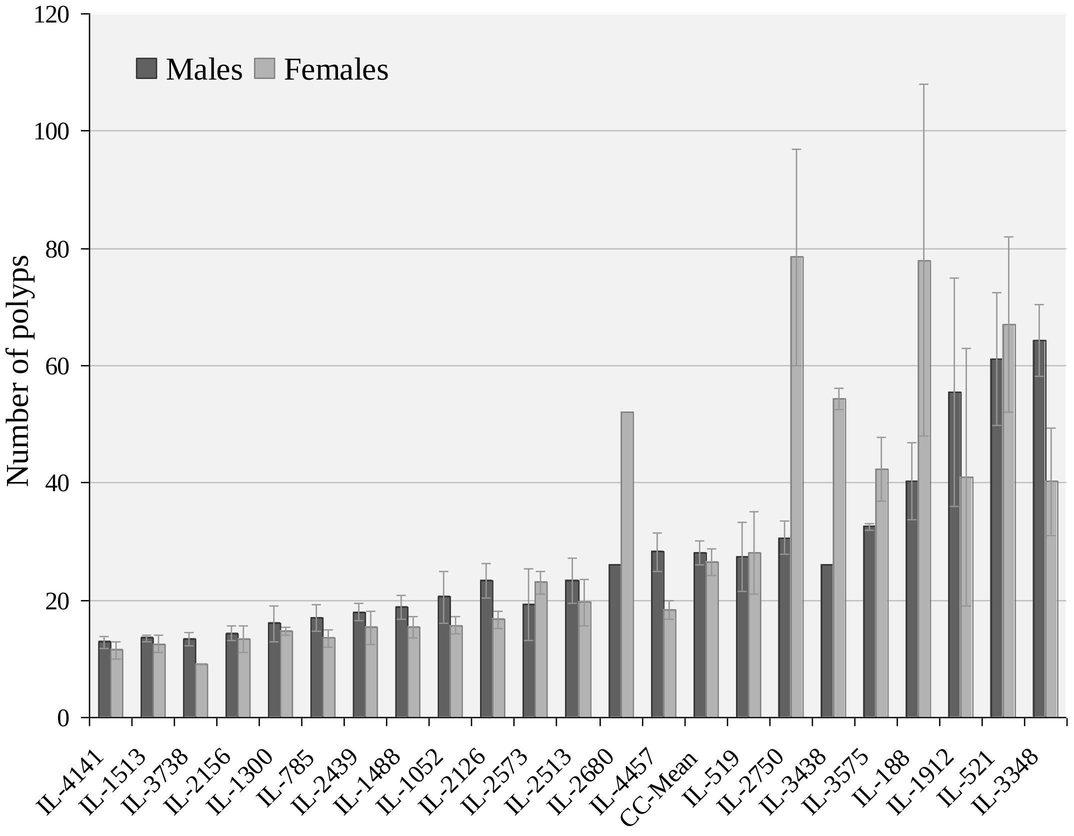

Supplement: Additional file 1: — Table 1S: Summary of all generated progeny, divided by gender & Min carriers. Figure 1S. Intestinal length (cm) (±SE). The X-axis represents CC lines, C57BL/6 strain carrying the Apc Min/+ mutation (first black column) and CC-Mean (black column ~ in the middle), while the Y-axis centimeter. One-way ANOVA performed for statistical analysis, p < 0.05. Figure 2S. Number of polyps per cm (±SE). The X-axis represents CC lines, C57BL/6 strain carrying the Apc Min/+ mutation (first black column) and CC-Mean (black column ~ in the middle), while the Y-axis is number of polyps. One-way ANOVA performed for statistical analysis, p < 0.05. Figure 3S. Frequency of lines as for the number of polyps per cm. The X-axis represents number of polyps per cm, while the Y-axis is the frequency. Number inside each column is the number of CC lines within each range of polyps. Figure 4S. Intestine %Weight (±SE). The X-axis represents CC lines, C57BL/6 strain carrying the Apc Min/+ mutation (first black column) and CC-Mean (black column ~ in the middle), while the Y-axis %Weight. One-way ANOVA performed for statistical analysis, p < 0.05. Figure 5S. %PCV (±SE). The X-axis represents CC lines, C57BL/6 strain carrying the Apc Min/+ mutation (first black column) and CC-Mean (black column ~ in the middle), while the Y-axis %. One-way ANOVA performed for statistical analysis, p < 0.05. Figure 6S. Spleen %Weight (±SE). The X-axis represents CC lines, C57BL/6 strain carrying the Apc Min/+ mutation (first black column) and CC-Mean (black column ~ in the middle), while the Y-axis %Weight. One-way ANOVA performed for statistical analysis, p < 0.05. Figure 7S. Average number of polyps (±SE) in 22 F1 CC-C57BL/6-Min lines (n = 1-16 mice/line) based on male and female. The X-axis represents male and female of each studied CC line, while the Y-axis number of polyps. (PDF 998 kb) [file 12863_2016_349_MOESM1_ESM.pdf]
